# How Does the Study MD of pH-Dependent Exposure of Nanoparticles Affect Cellular Uptake of Anticancer Drugs?

**DOI:** 10.3390/ijms24043479

**Published:** 2023-02-09

**Authors:** Selvaraj Sengottiyan, Alicja Mikolajczyk, Tomasz Puzyn

**Affiliations:** Laboratory of Environmental Chemoinformatics, Faculty of Chemistry, University of Gdansk, Wita Stwosza 63, 80-308 Gdansk, Poland

**Keywords:** cell uptake, drug-loaded nanoparticle, drug delivery, anticancer drug, MD

## Abstract

The lack of knowledge about the uptake of NPs by biological cells poses a significant problem for drug delivery. For this reason, designing an appropriate model is the main challenge for modelers. To address this problem, molecular modeling studies that can describe the mechanism of cellular uptake of drug-loaded nanoparticles have been conducted in recent decades. In this context, we developed three different models for the amphipathic nature of drug-loaded nanoparticles (MTX-SS-γ-PGA), whose cellular uptake mechanism was predicted by molecular dynamics studies. Many factors affect nanoparticle uptake, including nanoparticle physicochemical properties, protein–particle interactions, and subsequent agglomeration, diffusion, and sedimentation. Therefore, the scientific community needs to understand how these factors can be controlled and the NP uptake of nanoparticles. Based on these considerations, in this study, we investigated for the first time the effects of the selected physicochemical properties of the anticancer drug methotrexate (MTX) grafted with hydrophilic-γ-polyglutamic acid (MTX-SS-γ-PGA) on its cellular uptake at different pH values. To answer this question, we developed three theoretical models describing drug-loaded nanoparticles (MTX-SS-γ-PGA) at three different pH values, such as (1) pH 7.0 (the so-called neutral pH model), (2) pH 6.4 (the so-called tumor pH model), and (3) pH 2.0 (the so-called stomach pH model). Exceptionally, the electron density profile shows that the tumor model interacts more strongly with the head groups of the lipid bilayer than the other models due to charge fluctuations. Hydrogen bonding and RDF analyses provide information about the solution of the NPs with water and their interaction with the lipid bilayer. Finally, dipole moment and HOMO-LUMO analysis showed the free energy of the solution in the water phase and chemical reactivity, which are particularly useful for determining the cellular uptake of the NPs. The proposed study provides fundamental insights into molecular dynamics (MD) that will allow researchers to determine the influence of pH, structure, charge, and energetics of NPs on the cellular uptake of anticancer drugs. We believe that our current study will be useful in developing a new model for drug delivery to cancer cells with a much more efficient and less time-consuming model.

## 1. Introduction

**Figure 1:**
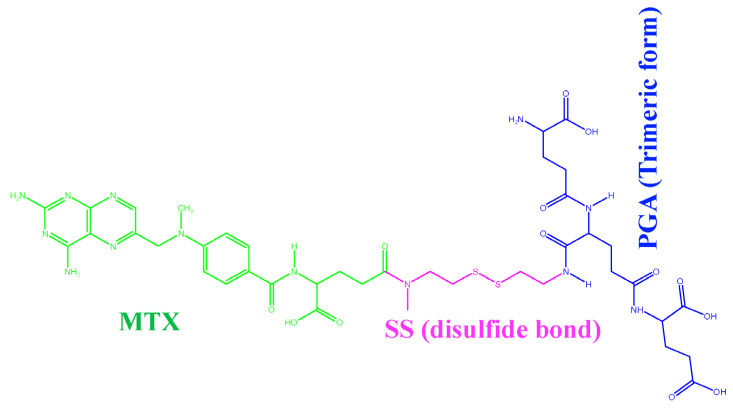
MTX-grafted disulfide-polyglutamic acid (MTX-SS-PGA).

In recent decades, the number of cancers in humans has increased significantly due to various factors and remains one of the second leading causes of death [1]. According to global statistics, there were 18.1 million new cases in 2018, with a mortality rate of 9.1 million, excluding skin cancer [2]. Solving this problem is a major challenge that can reduce the number of annual deaths. Although many chemotherapeutic agents are available, they cause serious side effects, lower therapeutic efficacy, and multidrug resistance [3,4]. Many conventional drugs are on the market to treat these diseases, but most of them have low efficacy. Due to low solubility, low bioavailability, and low efficacy in treating these factors, it is impossible to provide effective treatment for a long period of time [5]. To overcome this obstacle, nanotechnology plays an important role in targeted drug delivery throughout the body [6]. In this case, water-soluble drugs are poorly hidden in the micelles of drug-loaded nanoparticles using nanochemical methods and protected by a hydrophilic molecular fragment of the micelles in the external environment, resulting in a better ratio between the solubility of the drug and the water. This type of drug-loaded nanoparticle is sensitive to the external environment and helps to release the drug [7] at exactly the right location. In addition, the tailored design of smart nanoparticles (known as nanocapsules) is now widely used to solve this problem efficiently. Recent research has shown that paclitaxel [8] and doxorubicin [9] can be successfully developed as efficient anticancer drugs. The functionality of drug-loaded nanoparticles is also important for the development of potential medical imaging and therapeutic sensors [10]. Experimental and theoretical studies [11,12,13] have investigated the permeability of drugs in membranes. Distribution in a membrane is often studied using molecular dynamics simulations (MDS) [14,15]. Due to the high cost and potential risk to subjects involved in in vivo studies of intestinal membrane permeability, in vitro models, such as the distribution in isotropic systems [16,17], transport mechanisms with an artificial membrane [18], and a cultured monolayer of epithelial cells [19,20], have proven useful tools. However, these models do not take into account the molecular properties of the membrane, which play an important role in drug permeability. In addition, numerous studies have been conducted on the size, shape, and surface properties of nanoparticles [21] and their effects on the mechanism of cellular uptake. Many studies have identified the pH-dependent effect [22,23] of nanoparticle behavior on the cellular uptake mechanism through coarse-grained simulation studies [24], with a lack of degrees of freedom, charge, structure, and energy. However, systematic knowledge of the influence of physicochemical factors affecting the cellular uptake mechanism of drug-loaded NPs at different pH values is limited. Therefore, the present study aims to provide new insights into how the charge, structure, and energy of drug-loaded nanoparticles affect cellular uptake by different pH parameters. The case study was developed for the anticancer drug methotrexate disulfide polyglutamic acid (MTX-SS-PGA) loaded preparation based on three theoretical models for (1) pH 7.0 (so-called neutral pH model), (2) pH 6.4 (so-called tumor pH model), and (3) pH 2.0 (so-called stomach pH model). A cellular uptake mechanism was predicted using molecular dynamics simulations (MDS) based on extensive experimental evidence of efficient uptake by cancer cells [25]. Therefore, in this case, we modeled polyglutamic acid as a trimmer form to avoid the complexity of the atomistic simulation. The natural hydrophobic core of MTX is loaded/grafted with a disulfide bond nanocarrier containing hydrophilic glutamic acid, as shown in Figure 1.

## 2. Results and Discussion

### 2.1. Geometry Optimization and Drug Activity

**Figure 2:**
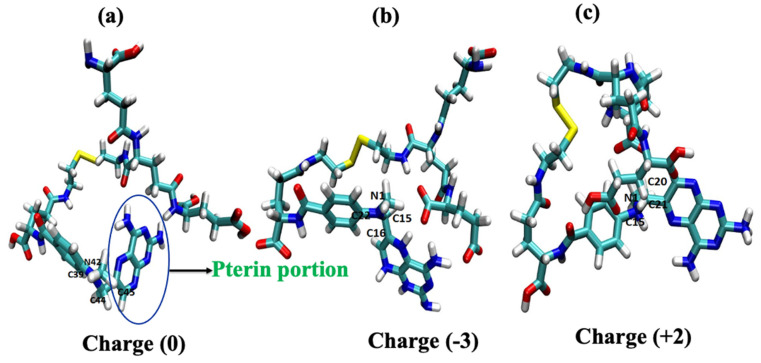
Geometry-optimized structural models of drug-loaded nanoparticles: (**a**) pH of ~7.0 (neutral model) and (**b**) ~6.4 (tumor model) and (**c**) ~2.0 (stomach model).

The geometrically optimized MTX-loaded nanocarrier molecules with three different models (the so-called neutral model, the tumor model, and the stomach pH model) of structural analogs determine the cellular uptake mechanism. Experimental results by Herd et al. [26] have shown that geometry is one of the most important criteria for cellular uptake. Our results for the neutral model show that the geometry is most likely to be cage-like (see Figure 2a). The other two simulated models also have similar cage-like orientations (see Figure 2b,c). Yating et al. [27] reported that a spherical nanoparticle is clearer and better taken up by cells than a rod-shaped particle; based on these observations, our models are cage-like, spherically symmetric nanoparticles that are more easily taken up by the cell membrane (CM). Thus, we can conclude that structural analogs are also important criteria for influencing cellular uptake mechanisms. Liang et al. [28] reported that the pterin moiety of the methotrexate drug plays a critical role in antimetabolite activity because it has a binding affinity to dihydrofolate reductase within the cell; when there is a change or substitution of any group in the pterin moiety, it decreases the drug activity. Based on this criterion, we hypothesize here that a change in the dihedral angle affects cellular uptake because the hydrophobic core of the pterin moiety readily penetrates the hydrophobic tail of the lipid membrane and also affects drug activity. The pterin ring is almost perpendicular to the drug molecule in the dihedral angle models (ɸ_1_=82.16) of the neutral model (C_39_-N_42_-C_44_-C_45_) and the dihedral angle (ɸ_2_ =96.42) of the tumor model (C_22_-N_1_-C_15_-C_16_) and the narrow linear ɸ_3_ =153.20 of the stomach pH model (C_15_-N_1_-C_20_-C_21_), as shown in Figure 2 and Table 1. From this observation, we can conclude that cell uptake is more favorable when the dihedral angle (ɸ) of the pterin ring is perpendicular to the drug molecule, whereas the drug delivery process begins when it becomes linear.

### 2.2. RMSD and RMSF Analysis

**Figure 3:**
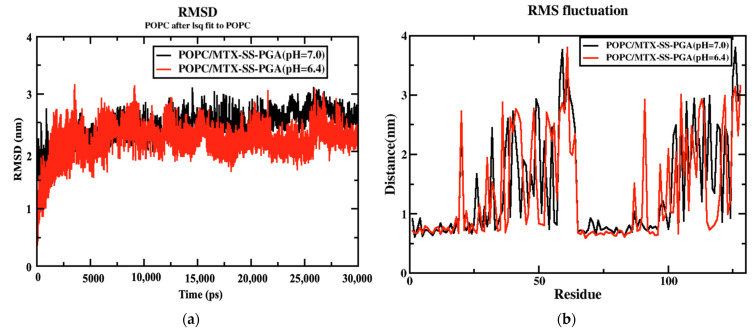
(**a**) RMSD diagram for both nanoparticles and (**b**) RMSF variation for both nanoparticles.

The coordinates of the two complex structures (POPC/MTX-SS-PGA) (pH = 7) and POPC/MTX-SS-PGA (pH = 6.4) of the simulation studies were compared with the original reference structure of coordinates. The derived root mean square deviation (RMSD) between the phosphate groups (PO_4_^3−^) of the membrane complexes with two models at 303.15 K showed that both systems reached equilibrium from the simulation at 1 ns up to 30 ns (see Figure 3a). However, in this case, the tumor model was more stable than the neutral model, indicating that the tumor model interacted strongly with the lipid bilayer. RMSF analysis of the two complexes showed a similar trend up to 30 ns (see Figure 3b). The fluctuation was lowest in the region between 60 and 80 residues, indicating that there was a strong interaction with the nanoparticles in this region.

### 2.3. Area Per Lipid

**Figure 4:**
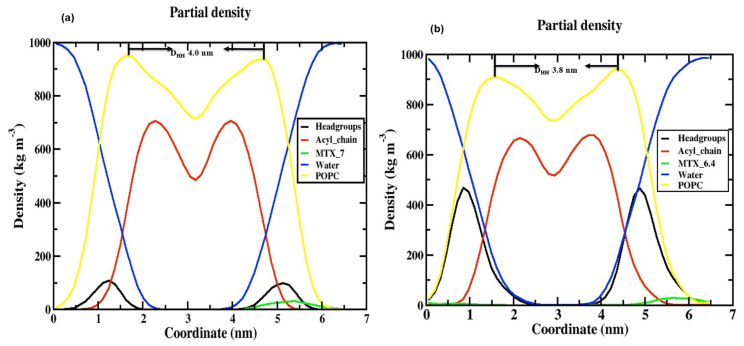
Electron density profile for various components in (**a**) POPC/MTX-SS-PGA (pH = 7.0) and (**b**) POPC/MTX-SS-PGA (pH = 6.4).

The area per lipid parameter is used to measure the compactness of the lipid bilayer and to determine whether additional permeation density affects the membrane. Tieleman et al. [29] reported that the calculated APL of the pure membrane is 65.8 Å^2^ and depends on the different temperatures: 66 Å^2^ at 310 K [30] and 62 Å^2^ at 310 K [31]. In our case, we observed the APL for two models of MTX-SS-PGA with pH values of 7.0 and 6.4 to be 63.94 Å^2^ and 63.89 Å^2^ at 313.15 K, respectively, as shown in Table 2 and Figure S1 (Supplementary Information). The third model, with a pH of 2.0, destabilizes [32] the system due to the high acidic pH of the medium during the MD production run, indicating that the drug delivery process has started; we could not determine the area per lipid for this model. In addition, the electron density for each lipid layer (POPC) refers to the electron-rich phosphorus group in the head group region present at the maxima of the peaks on both sides of the membrane, defined by the D_HH_ as the distance between the head-to-head maxima of the peaks. The D_HH_ for the tumor model was approximately 3.8 nm (slightly smaller than the experimentally found POPC membrane (39.1 Å)), and the neutral model was approximately 4.0 nm (slightly larger than the experimentally predicted POPC), as shown in Figure 4. This is due to the tumor model having a stronger interaction with the upper lipid bilayer than the neutral model due to the stronger electrostatic interaction of the charge variation of −3 for the tumor model and 0 for the neutral model. With this effect, the density of POPC in the tumor model (976.19 kg/m^3^) is slightly higher than that in the neutral model (963.93 kg/m^3^). Finally, the density was slightly impaired in both models compared with the POPC control. This suggests that our models did not have a large effect on the density of the membrane when passing through the cell membrane.

### 2.4. The Thickness of the Membrane 

The thickness of the membrane can be determined by quantifying the electron density profile using the X-ray scattering of a liquid crystalline lipid membrane [33,34]. The electron density profile is calculated by the following formula: electron charge = atomic number—partial electron charge at the center of an atom. The density profile is divided into different groups, such as POPC (1-palmitoyl-2-oleoyl-sn-glycerol-3-phosphocholine), phosphate (PO_4_^3−^), glycerol, water, MTX, and the acyl chain, as shown in Figure 4. Considering these two models, although both models show similar trend behavior, in the case of the density of the phosphate (PO_4_^3−^) group of the neutral (Figure 4a), there is a decrease (100 kg/m^3^) compared to the tumor pH model (Figure 4b) (475 kg/m^3^); this is due to the tumor model nanoparticles strongly interacting with the cell membrane at the top of the head group regions (NH_4_^+^) as the neutral pH model; this is due to the electrostatic interaction that occurs in the charge variation from neutral to tumor pH range (0 and −3 charge). In other areas, a very similar distribution was observed for both models (e.g., MTX, acyl chain, POPC, and water). In the middle of the inner bilayer, the density decreased, indicating the residence of the acyl chain of the phospholipid headgroups. The decrease in water density in the middle is due to the absence of water molecules in the middle bilayer. This indicates that no water molecules migrate through the lipid tail. In general, the density profile shows that the two models have a similar trend, except for the head groups. Our density curve shows that the parameters of our MD force field fit our problem well. However, in the case of the stomach, the pH model is destabilized during MD simulations because it is a highly acidic medium, as observed in this study [32]. The selection of the POPC membrane model is based on previous studies [35] on the role of anticancer drugs in the presence of cholesterol within the POPC membrane.

### 2.5. Order Parameters

**Figure 5:**
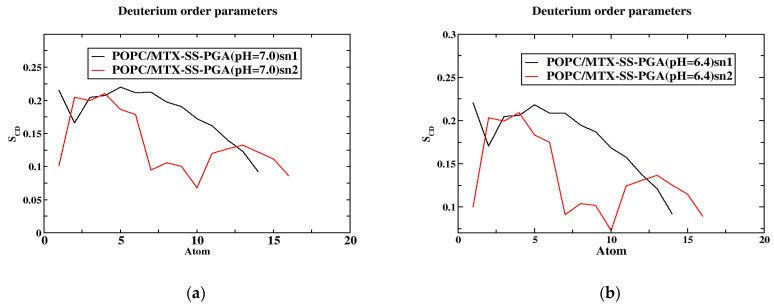
The order parameter of the SCD is a function of the position of the carbon atoms. The models (**a**,**b**) concerning the SCD of MTX-SS-PGA (pH = 7.0) and MTX-SS-PGA (pH = 6.4) in the sn1 and sn2 chains.

The order parameters were calculated for the POPC acyl chain in the presence of the tumor and neutral models and are shown in Figure 5. The results show that they agree well with previous simulations and experiments [36,37]. In both models, the order parameter of the C2 atom in both sn1 and sn2 is a deviation due to the orientation of the C2 chain in the acyl-chain region. The sn1 chains have a higher order parameter than sn2 because they are located near the head group of the phospholipid chain. The study by Vermeer et al. [37] showed that the S_CD_ of the C-D bonds is located near the head group, a similar behavior was observed in our MD simulations. Considering the unsaturated chain of POPC, the number of atoms 9 and 10 decreases due to the unsaturated bond in both models (see Figure 5a,b), but the tumor model decreases completely compared to the neutral model. Moreover, the high chain length of sn1 of the S_CD_ bonds gives more weight to the acyl chain than to the oleyl group of sn2 of POPC. The diagram clearly shows that the S_CD_ chain of the sn1 acyl chain is above ~0.2 in the near aqueous phase, as shown in Figure 5a,b). In the tumor model, the S_CD_ of sn1 is organized as a plateau from the first to the fifth segment, but in the case of the arrangement of sn2, the value of the S_CD_ bond has completely decreased, indicating that the acyl chain of the tail part decreases, and after the arrangement of the sixth carbon of sn1, the S_CD_ bond of ~0.175 decreases. In contrast, the sixth carbon in sn2 decreases again after a slight increase in the S_CD_ bond (~0.125) and the end of the carbon. A similar behavior is observed in the tumor model with maximum values of ~0.2 in the aqueous phase for the acyl chain, as well as in the neutral model. The minimum values of ~0.075 sn2 occurred in the tumor model, while they are almost zero in the case of the neutral model; this is due to the presence of a double bond [29]. The calculated values of the order parameters are in agreement with the theory and experiment. However, an error was found in S_CD_ due to the theoretical error limit in simulations compared to NMR spectroscopy methods.

### 2.6. Mean Square Displacement (MSD)

**Figure 6:**
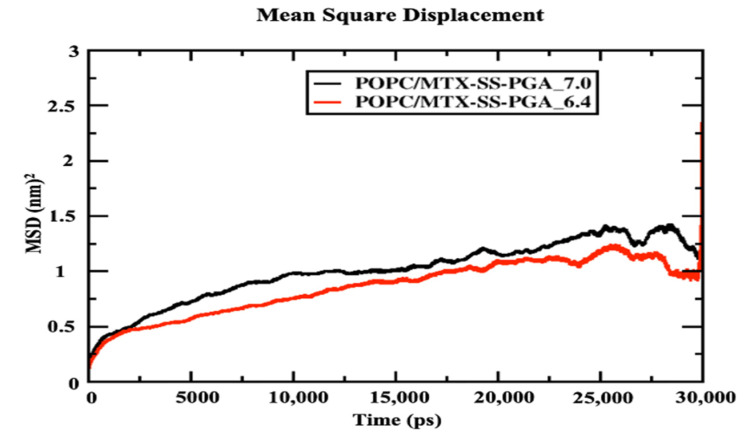
Mean square displacement of the center mass of POPC lipid leaflets with drug-loaded nanoparticles.

Figure 6 shows that the POPC bilayer with nanoparticles (MTX-SS-PGA (pH = 7.0) and MTX-SS-PGA (pH = 6.4) shows near linearity resulting from the entire simulation time, although the tumor model shows a decrease in the diffusion coefficient of 0.75 nm^2^ compared to the neutral model (1 nm^2^), suggesting that the condensation effect is due to the order [38] membrane. This is because the charge variation (−3) under this pH 6.4 tumor model strongly interacts with the top of the lipid bilayer, which leads to more order in the lipid bilayer than in the neutral model (the charge is zero). From this observation, our MD results show that the effect of lipid ordering is stronger in the tumor model than in the neutral model.

### 2.7. Potential Mean Force (PMF)

**Figure 7:**
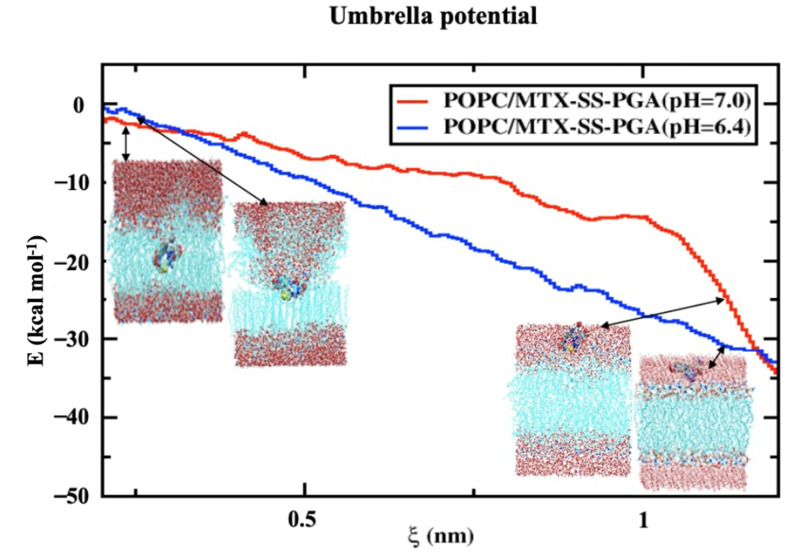
Free energy profile for the transfer of a nanoparticle loaded with a drug from the water phase to the center of the lipid bilayer. The reaction coordinates are shown from the center-of-mass of the drug-loaded nanoparticle to the center-of-mass of the lipid bilayer in the normal direction to the z-direction of the lipid bilayer interface.

The membrane of drug permeation studies can be determined in the solubility-diffusion relationship, although it is a critical calculation of the PMF curve, which is directly related to the relative solubility. In the case studied, we used two models of MTX-SS-PGA nanoparticles with two different pH values (7.0 and 6.4) to estimate the permeation/cell uptake of drug-loaded nanoparticles from the water phase to the membrane phase for tumor and neutral models, as shown in Figure 7. Considering the neutral model, it can be seen (Figure 7) that the free energy barrier from the water interface to the membrane is higher than the free energy barrier of the tumor model. This indicates that a nanoparticle with a pH of 7.0 is clearly preferable to a pH of 6.4 (shown in Supplementary Video S1). This result indicates that the bilayer permeation of the neutral model is preferred over that of the tumor model. Based on the inhomogeneous solubility-diffusion model [39] developed using the MD study, the permeation/cellular uptake of drug-loaded nanoparticles is one of the factors that depend on the solubility of the molecule in water, hydrophobic lipid environment, free energy values, and so on. Based on this assumption, the energy profile shows that the tumor model concepts have deep energy minima compared to the neutral ones, as shown in Figure 7, suggesting that passive transport is a simpler inhomogeneous solubility diffusion model, although both drug-loaded nanoparticles have a deep energy profile at the lipid bilayer interface. This could be due to the number of atoms present in the model. It could be mentioned that there were several difficulties in the calculation of the barrier, especially for the model with a neutral pH (shown in Supplementary Video S1), which could be due to the slow diffusivity of permeation through the lipid bilayer. Similar studies were performed by Frezard and Yacoub et al. [40,41], who attributed the passive transport mechanism to the presence of cholesterol, which can reduce the free area and volume of the liquid membrane, affecting drug transport. Both models (tumor and neutral) on the left side of the curve (Figure 7) clearly show that the higher energy barrier can be associated with the characterization of nanoparticle permeation into the hydrophobic tail of the bilayer core as higher in energy. As a result, the bulk region of the water phase has decreasing energy for both nanoparticles (MTX-SS-PGA pH (7.0 and 6.4)). Therefore, the free energy (ΔG) increases from the interior of the water surface to the membrane of the hydrophobic tail of the center of mass of the bilayer (COM). However, from the free energy profile, it is clear that nanoparticles with a tumor pH penetrate the bilayer more easily than neutral particles. There is a controversy; in this case, the difference in free energy (Figure 7) may be the cause of the increase in the head group of the density map, i.e., different domains at different locations. How does this affect the density profile of the different regions? In contrast, the tumor model (Figure 7) is easily taken up by the cell membrane, and the density map of the head group (Figure 5) is not well reduced. This might be due to a strong interaction with the lipid head groups because the charge of the model is −3, so the electrostatic interaction of opposite forces affects the density of the head groups. Instead, the neutral model interacted with the lipid bilayer headgroups, which is due to the amphiphilic nature of the nanoparticle. Therefore, the density-energy profile (head groups) is much more affected by the tumor model (Figure 4) than by the neutral model. In addition, the geometry of the structure is also important for cell uptake because, in the case of our models, a more cage-like structure penetrates more easily into the models without significant interference from the bilayer; consequently, even with energetic criteria, energy is the determining factor for the permeation mechanism of nanoparticles from the water phase into the membrane interior. In this context, cell uptake can affect the NP charge, structure, and energy criteria of NPs to allow the drug/nanoparticle to permeate from the water phase into the membrane.

### 2.8. Hydrogen Bonding and Radial Distribution Functions

**Figure 8:**
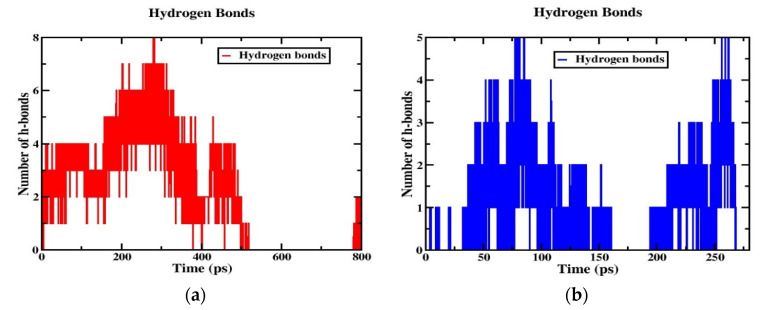
Hydrogen bonds for (**a**) POPC/MTX-SS-PGA (pH = 6.4) and (**b**) POPC/MTX-SS-PGA (pH = 7.0).

**Figure 9:**
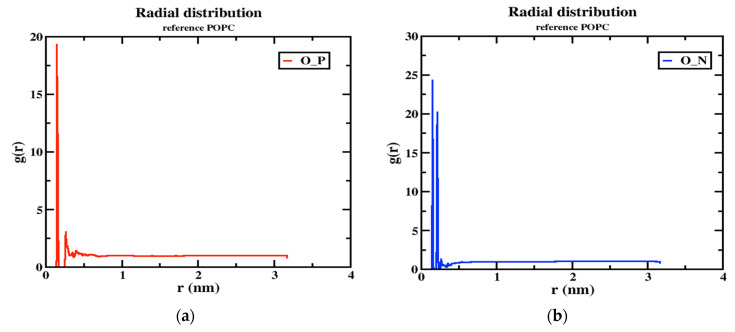

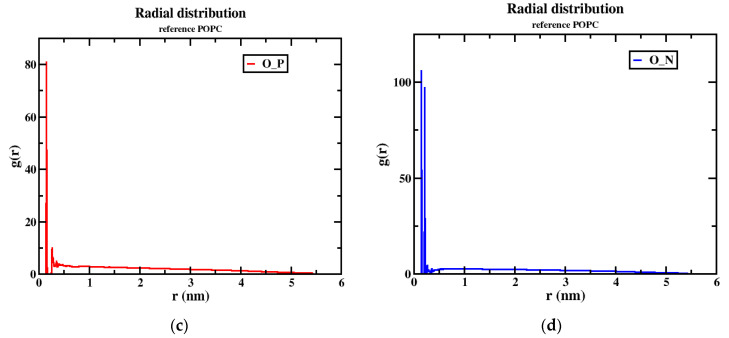
The RDF peaks for oxygen atoms of the nanoparticle with P and N atoms of POPC (1) MTX-SS-PGA (pH = 6.4) (**a**,**b**) (2) MTX-SS-PGA (pH = 7.0) (**c**,**d**).

Although the two models (neutral and tumor) have a cage-like structure, the cellular uptake of NPs differs because of the different structures, charges, and energetics of the NP system; based on these criteria, the tumor pH model penetrates the cell membrane faster than the neutral model. For this reason, the number of hydrogen bonds increased more in the tumor model than in the neutral model, indicating that the solubility ratio was higher in this model. The average number of hydrogen bonds of the tumor model is four within the time axis, but in the maxima at 8 h-bonds by 300 ps; in the case of the neutral model, the average of the number of h-bonds is three, and in the maxima at five by 100 and 250 ps, as shown in Figure 8a,b. The RDFs describe the probability of finding one particle to another with a distance between r and r + dr, where the bond distance is deeper at the transition of the nanoparticle from the aqueous phase to the membrane interior with solubility and distance criteria with the phosphate and nitrogen atoms of a lipid bilayer with oxygen atoms of the nanoparticle. In this RDF, the distance between two atoms, such as the “O” atom of the nanoparticle and the “P” and “N” atoms of the membrane interface, is uniquely described. In the tumor model, the two peaks show a higher probability by ~19.0 and ~3.0 of the distance of the O-P atoms in Figure 9a. In the other higher distribution possibility, with about ~25 and ~20 of the distance of O-N atoms in a higher likelihood ratio than the O-P atoms in Figure 9b, a similar trend was also observed for the neutral model (Figure 9c,d) nanoparticles. The value of a higher likelihood ratio of ~20.2 and ~3.8 for the spacing of the distribution peaks for the O-P bond and the two similar peaks appeared in the range of ~25.0 and ~21.0 for the distribution of the O-N bond ratio. From this, the presence of a higher solubility and distance ratio of nanoparticles to membrane head groups (P and N atoms) is understandable, indicating that both nanoparticles at pH 7.0 and pH 6.4 have stronger interactions with an upper layer of membrane head groups. Due to this stronger interaction of neutral and tumor pH nanoparticles with the membrane, the penetration force is ultimately possible, but in contrast, the solubility ratio of tumor pH nanoparticles would be higher than that of neutral ones, leading to the confirmed higher penetration force at the membrane interface.

### 2.9. Dipole Moment and FMO Analysis

In general, the magnitude of the dipole moment plays an important role in the solvation-free energy of an aqueous medium [42]. Based on this assumption, there is a significant difference in the magnitude of the dipole moments in all three models in our case. For example, the tumor pH model has a significantly higher dipole moment (Debye) of 32.5 D than the neutral and stomach pH models, which are given in Table 3 as 5.3 D and 28.8 D, respectively. Therefore, higher dipole moments have the possibility of a stronger free solvation energy with the aqueous phase because of the interaction of high preferential dipole moments with the environment. In this assumption, the tumor pH model may have a higher free solvation energy with water and thus has more stability while penetrating the cell membrane because this solubility criterion is also an important factor in deciding the cell uptake. The second place is the stomach pH model (destabilized by the high acidity of the medium), and the last place is the neutral model. From the frontier orbital, the chemical reactivity [43] and cytotoxicity [44] of the nanoparticles can be deduced. At pH 7.0, the orbital HOMO is delocalized in the phenyl ring part of methotrexate (MTX) and the LUMO in the pterin ring part of MTX with the transition of π→π*(HOMO-LUMO), but in contrast, the transition of π→σ* is the localization of the HOMO-1-LUMO+1 delocalized orbitals at the pterin ring for HOMO-1 and the LUMO+1 at the disulfide bond, shown in Figure S2 (Supporting Information); in the case of the tumor pH model, it localizes the HOMO-LUMO transition of n→π* at the “C=O” group of a glutamic acid moiety at HOMO orbitals and the pterin ring at the LUMO orbital. Similarly, the same transition (n→π*) was observed at HOMO−1-LUMO+1, as shown in Figure S3, in the stomach pH model, with the “C = O” group of the glutamic acid moiety delocalized at the HOMO orbital and the LUMO shown orbitals of the pterin ring of the MTX moiety delocalized at the n→π* transition (HOMO-LUMO), but the σ→π*(HOMO-1-LUMO+1) transition observed in the σ-orbital at the disulfide bond and in the π*-orbital at the pterin ring is shown in Figure S4. Taking into account the energy gap, the tumor pH model shows a very low energy gap (0.06 eV) at the HOMO-LUMO transition, indicating a higher chemical reactivity with a highly potent cytotoxic effect [44] than another model, such as neutral (0.13 eV) and the second highest chemical reactivity of the energy gap (0.09 eV) in the pH 2.0 model; the values are listed in Table 3.

### 2.10. Implications for the Results

**Figure 10:**
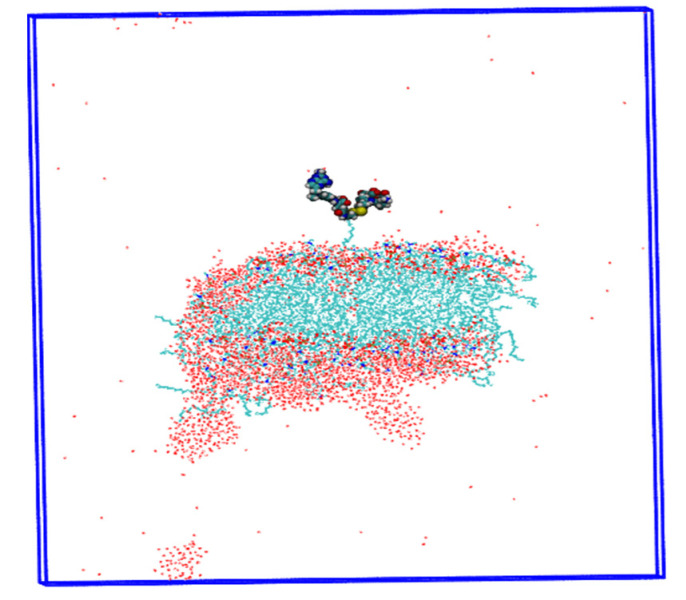
Insights into the drug delivery process are shown by the destabilization of the MTX-SS-PGA nanoparticle (pH = 2.0).

The results of Ningning Ma et al. [21] suggested that the cellular uptake of nanoparticles is influenced by size, shape, and surface area in previous reports. In addition, Behzadi et al. [45] suggested that variation in the colloidal stability of nanoparticles in biological media is also an important factor in discriminating cellular uptake rather than size, surface area, or surface charge (ζ). Therefore, we propose different parameters of NPs, such as charge, structure, and energy, calculated for three models with different pH values. In the third model of the stomach pH range, drug-loaded nanoparticles release the drug at this time, while the glutathione enzyme breaks the disulfide bond (MTX-SS-PGA) and begins to release the anticancer drug, as experimentally demonstrated [25]. This effect could be related to a more acidic environment [32], leading to the destabilization of the system during the production step (Figure 10). As a result, the nanoparticles begin to release the drug. The uptake in the tumor model is much shorter than in the neutral model. These results may be useful for developing a new methodology for drug development using a nanoparticle carrier medium.

## 3. Computational Methods

### 3.1. Case Study with Molecular Dynamics (MD) Simulations

**Figure 11:**
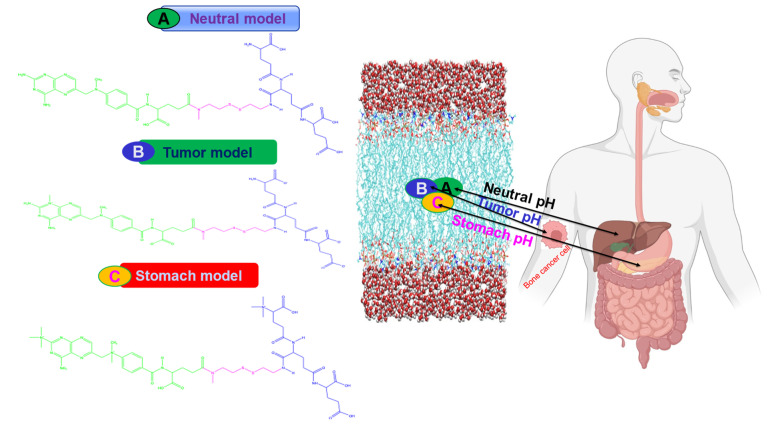
Schematic representation of the different models of the cellular uptake mechanism.

In the present study, we developed three different models of drug-loaded nanoparticles (MTX-SS-PGA) that respond to the pH of different organ sites (Figure 11). The first model was for pH 7.0 (the so-called neutral pH model), the second model was for pH 6.4 (the so-called tumor model), and the third model was developed for pH 2.0 (the so-called stomach pH model).

For these three models, the uptake and knowledge of the delivery process were obtained by fully atomistic MD simulations. In the present study, we hypothesize that the three different models of charge, structural analogs, and energetics strongly influence the cellular uptake mechanism at different pH media intervals. The models developed (Figure 11) will be optimized with a full atomic method to capture the cellular uptake and release process at different organ sites in the human body. From the barrier analysis, among the three models, the pH of the tumor (~6.4-mouth pH range) in which cells are taken up is faster than the neutral pH of the model, which is confirmed by the PMF curve obtained from the umbrella sampling and WHAM analysis methods. This will help us to understand the mechanism of drug-loaded nanoparticles with cell behavior in the human body. From this point of view, the third model with stomach pH (2.0) was used for drug delivery based on the results of MD simulations. In this case, the model is destabilized because the acid-labile [32] environment of drug-loaded nanoparticles is less stable in an acidic environment. Most importantly, our computational studies will be useful for predicting the mechanism of cellular uptake of drug-loaded nanoparticles and for providing useful insights into the drug delivery process.

### 3.2. Generation of the Models and Parameterization

The first neutral pH model describes the neutral pH model, which may represent parts (organs) of the human body, such as the liver (pH ~7.0). In most cases, the neutral pH model is less taken up by the cell membrane than by the ionic states of the particles, especially the anionically charged particles [46,47,48]. The second tumor pH (tumor model) describes the anionically charged particles (−3), which have the highest affinity [49] for the CM and consequently are captured by the CM with a higher order of magnitude of efficiency, although with lower uptake, than the cationically charged particles (third model). The anionic surface-charged particles significantly reduced the non-specific interaction with the plasma membrane and the overall uptake of CM, while restoring the ability to induce the specific interaction at the target cells. The third model of the stomach pH model of positively charged (+2) particles have the highest uptake compared to neutral and negatively charged particles [50,51] due to higher internalization with CM and because negatively charged CM has a strong affinity for positively charged particles. The two models (tumor and stomach) were created using the dimorphite-DL [52] program under the control of the neutral model. The program provides SMILE formatted structural information with the corresponding pH values, and on this basis searches the pKa values from the database to generate at the end all possible conformations of the structural analogs in the form of a SMILE formatted file considering the pH value of the molecule, once we obtained a molecular geometry optimized with the B3LYP exchange-correlation function of the 6-31G* basis set using the DFT theory of the Gaussian 09 package [53]. Using this optimized geometry of nanoparticles, the RESP charge was generated based on HF/6-31G* using GAMESS-US [54]. The optimized geometries of the three different pH models were confirmed with energy-minimized conformations by vibrational analysis without imaginary frequency.

### 3.3. MD Setup

Three models of MD simulation, (1) a pH~7.0 of drug-loaded nanoparticles, (2) a pH~6.4, and (3) a pH~2.0, consisting of a classical POPC membrane with 64 lipids per leaflet, were performed using a CHARMM-GUI [55] membrane builder with a force field of CHARMM36 FF [56] for lipids. All simulations were performed using a TIP3P water model with additional CHARMM36 force field parameters. The generated model was solvated with TIP3P [57] water molecules on total charge replaced by counterions. Neutralities of POT+ and Cl- were added to the membrane composition for each simulation box.

### 3.4. Equilibration and Production Setup

Each structural model was placed in this solute membrane simulation box and equilibrated with different types of equilibration steps using a multistep protocol [57]. Seven different types of short-term equilibration steps were performed with different thermostat conditions, force constants with different position-limited values, and others to maintain the stability of the membrane and allow the water molecules to equilibrate. In the equilibrium step, the pressure (P = 1 atm) and temperature (T = 303.15 K) were kept constant by maintaining a damping force coefficient value of 0.5 ps^−1^ with a piston start-up time of 50 fs. At the same time, the Berendsen thermostat [58] was used for pressure control and subsequent equilibrium steps with a semi-isotropic Noose–Hoover scheme [59] to control the pressure at the thermostat with a piston drop of 25 fs. The simulation setup of 2 fs used the integrated equation of motion Verlet algorithm [60]. An isothermal-isobaric ensemble (NPT) was used for the periodic condition. The LINCS [61] constraint algorithm was applied to all h-bond involving atoms. The particle mesh Ewald (PME) method [62] was used to extract all long-range electrostatic interactions with a spatial cut-off point of 12 Å. The Lennard–Jones interactions were applied to a cut-off value of 10 Å, which is further truncated to 12 Å. A SHAKE [63] algorithm was applied under a holonomic constraint to capture all covalent bonds associated with hydrogen atoms. MD simulations and analyses were performed using the Gromacs 2020.2 software package [64].

### 3.5. Potential Mean Force (PMF) Simulations

The umbrella sampling approach was used to calculate the potential mean force of the free-energy profile for these models. Calculating the partitioning of the three models between the membrane and the surrounding water is also possible. For the three models of the upper layer in the aqueous phase of water at a tensile force of 1000 kJ/mol·nm^2^ with a tensile speed of −0.2 nm/s, an attempt was made to pull the drug-loaded nanoparticles toward the center of the membrane by a total of 30 Å in the scheme of the semi-isotropic NPT ensemble. During the simulation time, the snapshots from the top (z = 30) to the center (z = 0) were saved every 1 Å interval time, with a total of 30 windows to calculate the PMF curve. Because of the symmetrical nature of the bilayer, the remaining half of the distance reflects the same. Starting from this point, the reflectance data along the z-axis of the windows around 30 and 29 in the middle of the double layer are the same for the remaining distance. In each window, the production run was performed up to 1 ns, with a force constant of 1000 kJ/mol·nm^2^ with a total sampling of 1 μs of the nanoparticles entering the membrane. During the simulation, the configurations were saved every 1 ps; at the end, WHAM [65,66] analysis was used to combine the biased, distributed, and reweighted analysis to calculate the PMF curve.

### 3.6. Area Per Lipid (APL)

The area per lipid analysis was used to extract the molecular packing of the lipid bilayer. The value of APL provides information about the arrangement of lipids and the structural and dynamic properties of the membrane. When calculating the APL, the normal bilayer is considered along the z-axis. The APL can be calculated using Equation (1):

(1) $$\mathrm{APL}=\frac{L_{x}{\;L}_{y}}{{\;N}_{\mathrm{lipid}}}$$

Here, L_x_ and L_y_ are the lengths of the box direction in x and y. N_lipid_ indicates the number of lipids present in one leaflet.

### 3.7. Order Parameters

The order parameter S_CD_ can determine the lipid acyl chains present in the lipid bilayer, and the amount of S_CD_ can easily be compared with the experimental S_CD_ values from ^2^H NMR and ^1^H-^13^C NMR. This allows the relative orientation of C–D relative to the normal bilayer to be calculated. This can be determined by the following Equation (2).

S_CD_ = 0.5 <3cos2θ − 1> (2)

where θ indicates the angle between the normal bilayer and vector joining C-D (in our case, C-H in the simulation); <> determines an average ensemble. 

### 3.8. Lateral Diffusion Coefficient

Lateral diffusion is used to measure the ability of lipids to move through leaflets; it is an important dynamic property to be characterized and measured by the diffusion coefficient derived from Equation (3).

D(τ) = lim_τ→∞_ (MSD(τ)/4τ) (3)

where τ is the elapsed time and MSD is the mean square displacement of the selected lipids in the center of the mass of the XY plane, averaged over the different initial times and the number of lipids.

## 4. Conclusions

In this work, we address the problem of cell uptake by drug-loaded nanoparticles predicted by MD simulations, suggesting that the tumor pH model is easier to uptake than the neutral model. The influence of cellular uptake by the NP’s charge, structure, and energetics of NP is based on the system. However, the stomach pH model begins to deliver the drug in a more acidic environment. In a more acidic environment, the nanoparticle was not more stable under these conditions during the production step, which was verified by MD trajectories. In our case, we found that the permeation of drug-loaded nanoparticles did not affect the membrane architecture. The analysis of the APL, density, thickness, and order parameters imparts a similar trend of convergence, although they vary with the pH model. The observation of the PMF curve indicates that the tumor pH model has a lower energy barrier than the neutral model, confirming that drug-loaded nanoparticles cross the membrane easily. Density analysis shows that the peak of the phosphate headgroup is more reduced in the neutral model than in the tumor model. This means that the tumor pH nanoparticles have stronger interactions with the head group (NH_4_^+^) of the cell membrane. In contrast, the neutral model has neutrally charged (0 charge) nanoparticles that do have possibly less interaction with the headgroup region of the cell membrane maybe due to less electrostatic force. Furthermore, the mechanism of cellular uptake is elucidated by the hydrogen bonding analysis, RDF. It was found that the tumor model has more hydrogen bonds with water and the membrane, which can be more easily taken up by the cell membrane than the neutral model. The dipole moment and HOMO-LUMO analysis show that the magnitude of the dipole moment can have a free solvation energy with the water phase as a result of the polarizing force with the environment. According to our analysis, the tumor pH has a high dipole moment of 32.5 D compared to other models, which will ultimately affect the penetration forces within the cell membrane. HOMO-LUMO, the analysis shows us the nature of the orbital transitions in the ground state geometry, which could indicate the delocalized charges where they are placed within the molecule. Based on this, we could express the bioactivity of the molecules. In this context, tumor pH has a very low energy gap, which is less stable and has high chemical reactivity than other models, but both transitions (n→π*) are the same (HOMO-LUMO and HOMO-1-LUMO+1); secondly, it has a lower energy gap of the pH 2.0 model and, finally, the neutral one. This analysis suggests that all three models have high chemical reactivity, possibly exceeding the interaction of nanoparticles with the cell membrane, and that remarkable cytotoxicity is always hindered due to this high potency in acidic environments, such as the pH range of the tumor and the stomach. Our results are consistent with the experimental methods for the neutral model; drug-loaded nanoparticles were found to be highly capable of pH-dependent cellular uptake by the NP criteria of charge and geometry, and the energy-dependent internalization of the mechanism was observed. In this context, we propose a mechanism of action when these models penetrate the cell membrane by using our observation of the subtle importance of cell-penetrating drug-loaded nanoparticles in interaction studies with the lipid bilayer. However, our proposed results show that these methods are useful for designing different modeled drug-loaded nanocarriers with different concentrations and pH values, which will be more useful for future research on cancer treatment.

## Figures and Tables

**Table 1:** Charge and dihedral angles of the models (drug-loaded nanoparticles).

pH	Charge	Dihedral Angle (ɸ)
7.0	0	82.16
6.4	−3	96.42
2.0	+2	153.2

**Table 2:** Comparison of the structural parameters of the two models at the end of the MD simulation.

Lipid Membrane	POPC/MTX-SS-PGA (pH = 7.0)	POPC/MTX-SS-PGA (pH = 6.4)	POPC (Exp)
Time	30 ns	30 ns	
Area per lipid (Å^2^)	63.94 ± 0.01	63.89 ± 0.01	65.8 (Å^2^)
Density (kg/m^3^)	963.93 ± 0.001	976.19 ± 0.001	39.1 (Å) [33]

**Table 3:** The properties of dipole moment and energy gap (HOMO-LUMO) for models.

pH	Dipole Moment (Debye)	Energy Gap (∆E_H-L_)
7.0	5.3	0.13 eV
6.4	32.5	0.06 eV
2.0	28.8	0.09 eV

## Data Availability

Not applicable.

## Supplementary Materials

The following supporting information can be downloaded at: https://www.mdpi.com/article/10.3390/ijms24043479/s1.
