# Supplementary material for: How Does the Study MD of pH-Dependent Exposure of Nanoparticles Affect Cellular Uptake of Anticancer Drugs?
Source: Int J Mol Sci. 2023 Feb 9;24(4):3479. doi: 10.3390/ijms24043479 (PMC9958846; doi:10.3390/ijms24043479)

International Journal of
Molecular Sciences

MDPI

Article

How Does the Study MD of pH-Dependent Exposure of Nanoparticles Affect Cellular Uptake of Anticancer Drugs?

Selvaraj Sengottiyan, Alicja Mikolajczyk * and Tomasz Puzyn *

Laboratory of Environmental Chemoinformatics, Faculty of Chemistry, University of Gdansk, Wita Stwosza 63, Gdansk, 80–308 Poland
* Correspondence: alicja.mikolajczyk@ug.edu.pl (A.M.); tomasz.puzyn@ug.edu.pl (T.P.)

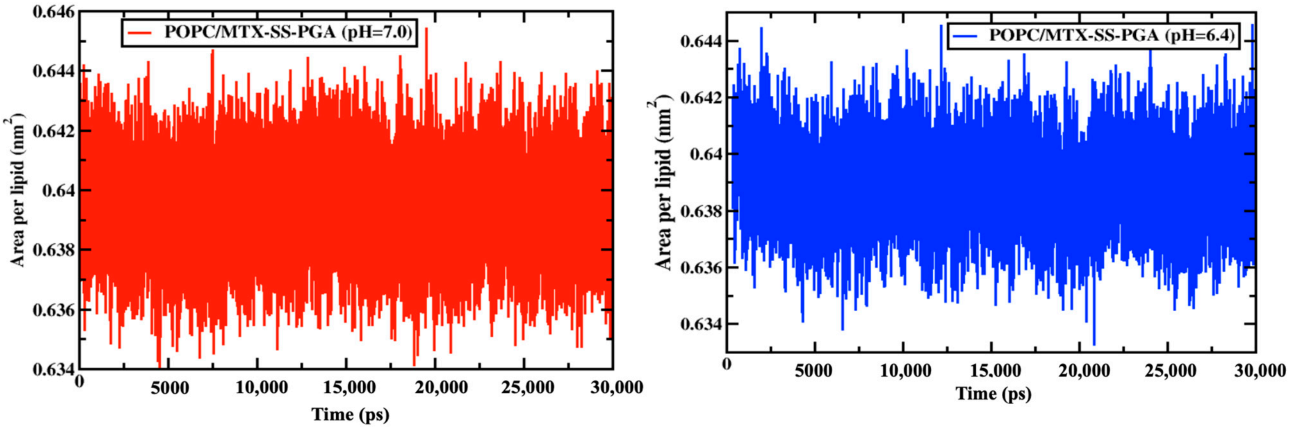

Figure S1. Area per lipid for (a) POPC/MTX-SS-PGA (pH=7.0) (b) POPC/MTX-SS-PGA (pH=6.4).

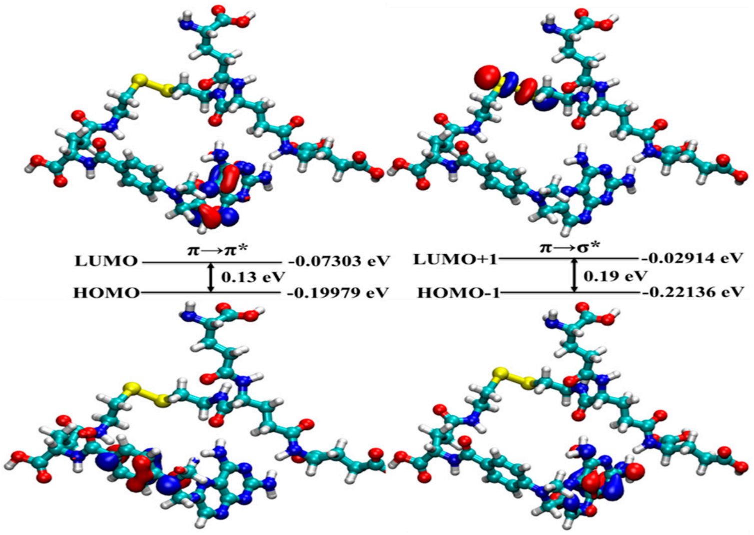

Figure S2. FMO analysis for POPC/MTX-SS-PGA (pH=7.0).

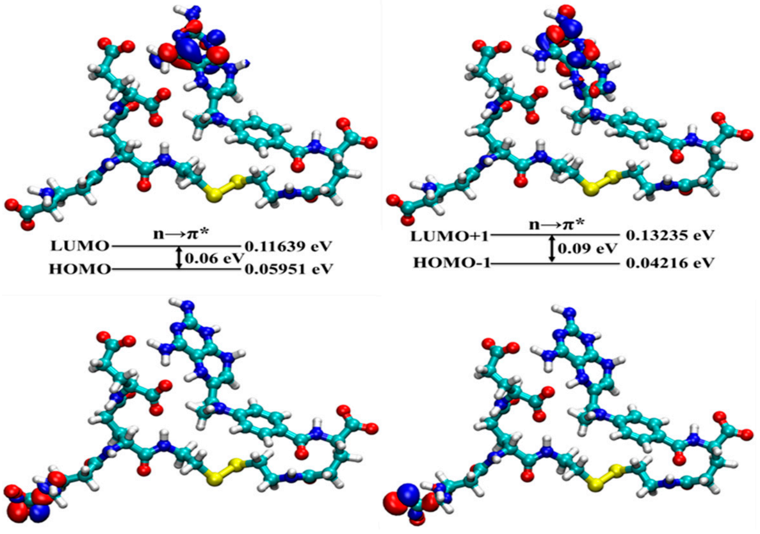

Figure S3. FMO analysis for POPC/MTX-SS-PGA (pH=6.4).

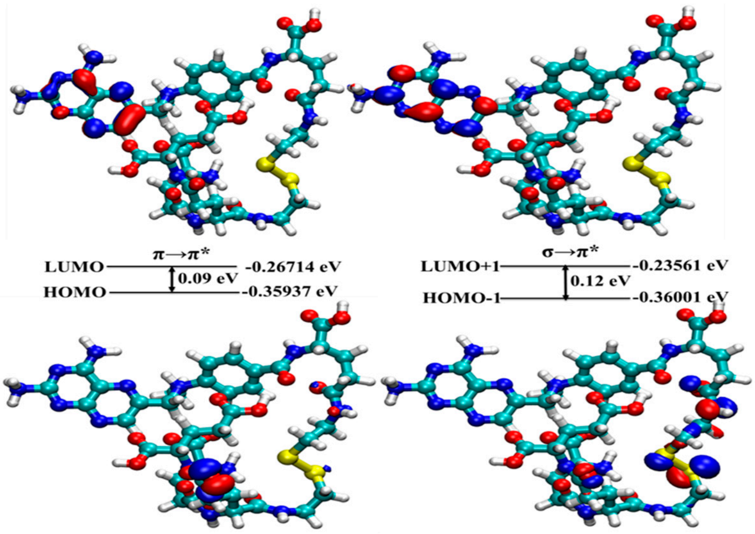

Figure S4. FMO analysis for POPC/MTX-SS-PGA (pH=2.0).

Supplementary video: S1

A movie illustrates the passage of nanoparticles of (a) MTX-SS-PGA (pH=7.0) and (b) MTX-SS-PGA (pH=6.4) into the lipid bilayer.

(a) (b)

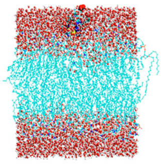

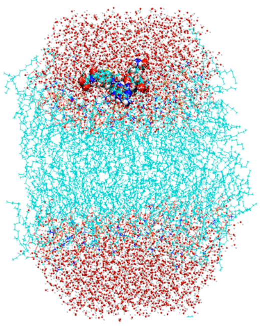

Supplement: Supplementary file 1 [file ijms-24-03479-s001.zip › ijms-2174799-supplementary.pdf]
